# Supplementary material for: A Vaccinia-based system for directed evolution of GPCRs in mammalian cells
Source: Nat Commun. 2023 Mar 30;14:1770. doi: 10.1038/s41467-023-37191-8 (PMC10063554; doi:10.1038/s41467-023-37191-8)
Supplement: Supplementary file 1 — Supplementary Information [file 41467_2023_37191_MOESM1_ESM.pdf]

Supplementary Information

A *Vaccinia*-based system for directed evolution of GPCRs in mammalian cells

Christoph Klenk, Maria Scrivens, Anina Niederer, Shuying Shi, Loretta Mueller, Elaine Gersz, Maurice Zauderer, Ernest S. Smith, Ralf Strohner, Andreas Plückthun

* Corresponding authors. Email: c.klenk@bioc.uzh.ch, plueckthun@bioc.zh.ch

Supplementary Notes

Tailored Library Design

For directed evolution of larger proteins such as GPCRs error-prone PCR is commonly used to introduce mutations. While this has proven to work for a number of receptors[1-6], some limitations of this technique must be considered. Unbiased randomization will inevitably lead to incorporation of unwanted mutations such as premature stop codons, the stochastic nature of error-prone PCR makes adjacent three-base changes extremely unlikely, limiting the accessible mutation through the biasing of the genetic code, and tuning the mutational load over the whole gene is difficult. Moreover, it has become increasingly clear that contacts within and between the transmembrane helices contribute most to receptor stability and function, and such residues can now be easily inferred from available protein structures or homology models. Therefore, a more directed design for GPCR libraries becomes possible which will improve the outcome of directed evolution.

To implement this, we applied a semi-rational approach for a tailored library design. For NTR1, the crystal structure of the thermostabilized variant NTR1-TM86V (PDB ID: 4BUO) was used to design the positions of mutations, but the only mutation permanently encoded in the library was R167$^{3.50}$L (see below). Residues that are facing into the receptor transmembrane bundle, and thus most likely contribute to allosteric interactions within the receptor core, were selected for randomization. Residues facing into the lipidic environment, or constituting the orthosteric binding pocket, or likely being involved in G protein interaction, were excluded. As a result, 94 positions were assigned for randomization.

Next, a GPCR-specific evolutionary substitution matrix was created which resembled commonly used matrices that are based on the frequencies of amino acid substitutions observed in aligned protein sequences[7-9]. For this purpose, a multiple sequence alignment from 296 class A GPCRs (excluding olfactory receptors) was obtained from GPCRdb, and the five most frequent amino acids at each position were determined to serve as library members for subsequent randomization (**Supplementary Fig. 1**). Notably, the structural template NTR1-TM86V contains 11 stabilizing mutations. With the exception of R167$^{3.50}$L (c.f. main text), none of these mutations were permanently encoded into the library.

For PTH1R, a slightly modified strategy was applied (**Supplementary Fig. 4**). Considering the complex multi-domain structure, inherent in class B GPCRs, randomization was restricted exclusively to the TMD, thereby omitting alterations to the ECD which primarily would affect full-length ligand binding. To identify residues for randomization, a homology model based on the structure of glucagon receptor (PDB ID: 4L6R) was used, since at the time, no structure for PTH1R had been available. Then, by applying the same algorithm as for NTR1, 99 positions were assigned for randomization. In contrast to the class A of GPCRs, the group of class B receptors is relatively small, and hence instead of an evolutionary model as for NTR1 we used a substitution matrix based on amino acid similarity (**Supplementary Fig. 4B**). In addition, we assigned 19 positions that had been evolved in a previous directed evolution approach of PTH1R in yeast[3] (Klenk et al. unpublished). For those residues, the same randomization matrix was applied, yet specific mutations, which had been identified in the yeast selection but were not included in the substitution matrix, were added manually (e.g. M312K, K359N, Q440R; **Supplementary Fig. 4C**).

To enable site- and sequence-specific randomization, cDNA libraries for NTR1 and PTH1R were created by Slonomics™ solid phase synthesis[10-12]. Binomial distribution was set to 3-5 mutations per gene, requiring a mutagenesis rate of ~1% for each of the 5 substituents.

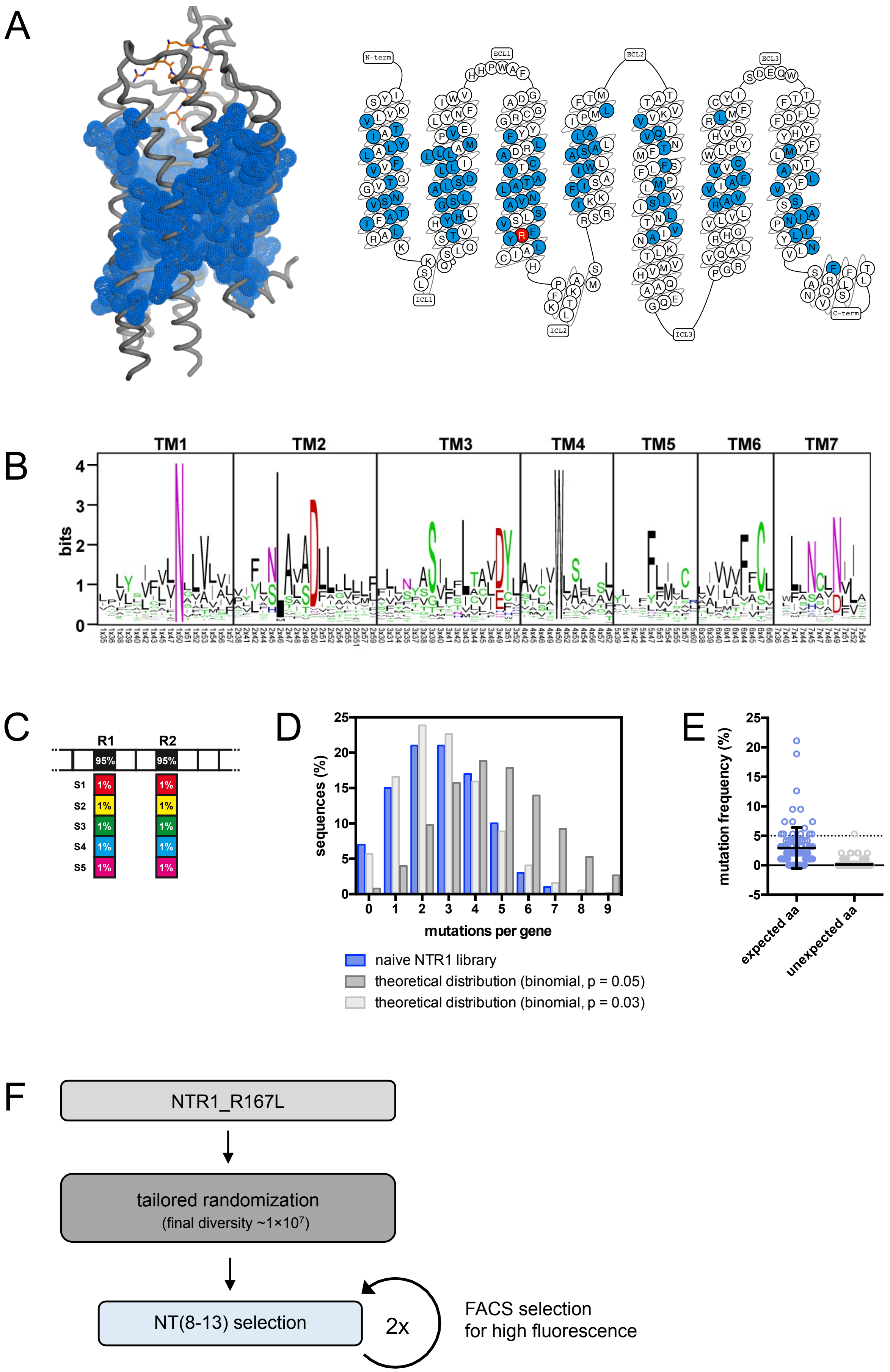
A
N-term
ECL1
ECL2
ECL3
ICL1
ICL2
ICL3
C-term
B
TM1
TM2
TM3
TM4
TM5
TM6
TM7
bits
C
R1
R2
95%
S1
S2
S3
S4
S5
1%
D
sequences (%)
mutations per gene
naive NTR1 library
theoretical distribution (binomial, p = 0.05)
theoretical distribution (binomial, p = 0.03)
E
mutation frequency (%)
expected aa
unexpected aa
F
NTR1_R167L
tailored randomization
(final diversity ~1×10^7)
NT(8-13) selection
2x
FACS selection
for high fluorescence

Supplementary Figure 1. Library design and selection strategy for NTR1. (*A*) Based on the crystal structure of rNTR1 (PDB ID: 4BUO), 94 residues were selected for randomization (blue). R167$^{3.50}$L was included as a fixed mutation to the library (red). (*B*) Substituents were selected based on an evolutionary substitution matrix. For this purpose, a multiple sequence alignment from 296 class A GPCRs (excluding olfactory receptors) was obtained, and the five most frequent amino acids at each position were determined to serve as library members for subsequent randomization. (*C*) A mutation frequency was chosen to yield an average number of 3–5 mutations per gene. For this purpose, per residue each of the 5 substituents was introduced with 1% frequency. (*D–E*) Mutation analysis of naïve NTR1-library. Sequence analysis of 95 clones of the naïve library reveals an average distribution of 2.8 mutations per gene, corresponding to a theoretical mutation rate of 3% instead of the anticipated 5% (*D*). At each position on average 2.93% residues corresponded to expected mutations whereas 0.13% were unexpected mutations (i.e. mutations at constant positions or non-encoded mutations). Each data point depicts the frequency of expected (encoded by the nucleotide triplets) and unexpected mutations (i.e., mutations at constant positions or non-encoded mutations) at a specific position of NTR1 (amino-acids 43–424). Dotted line, overall anticipated mutation frequency. Error bars represent s.d. (*E*). (*F*) Selection scheme for NTR1. After incorporating the library into the *Vaccinia* vector, A-431 cells were infected and 2 consecutive selection rounds with 40 nM HL647-NT(8–13) were performed.

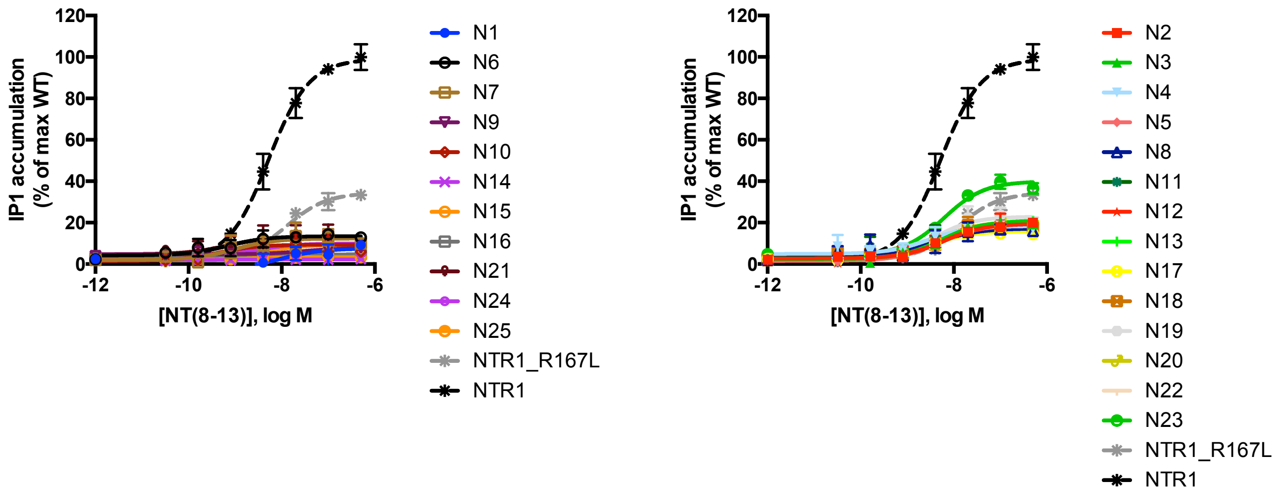

Supplementary Figure 2. Signaling activity of NTR1 mutants. For each receptor variant, G_q-mediated IP1 accumulation was measured in HEK293T cells after stimulation with NT(8–13). Left panel: variants exhibiting < 10% E_{max} of WT, right panel: variants exhibiting ≥ 10% E_{max} of WT. Data were normalized to IP1 levels of NTR1 wild type at 500 nM NT(8–13) and are shown as mean values (± s.e.m.) of 2 independent experiments each performed in duplicates.

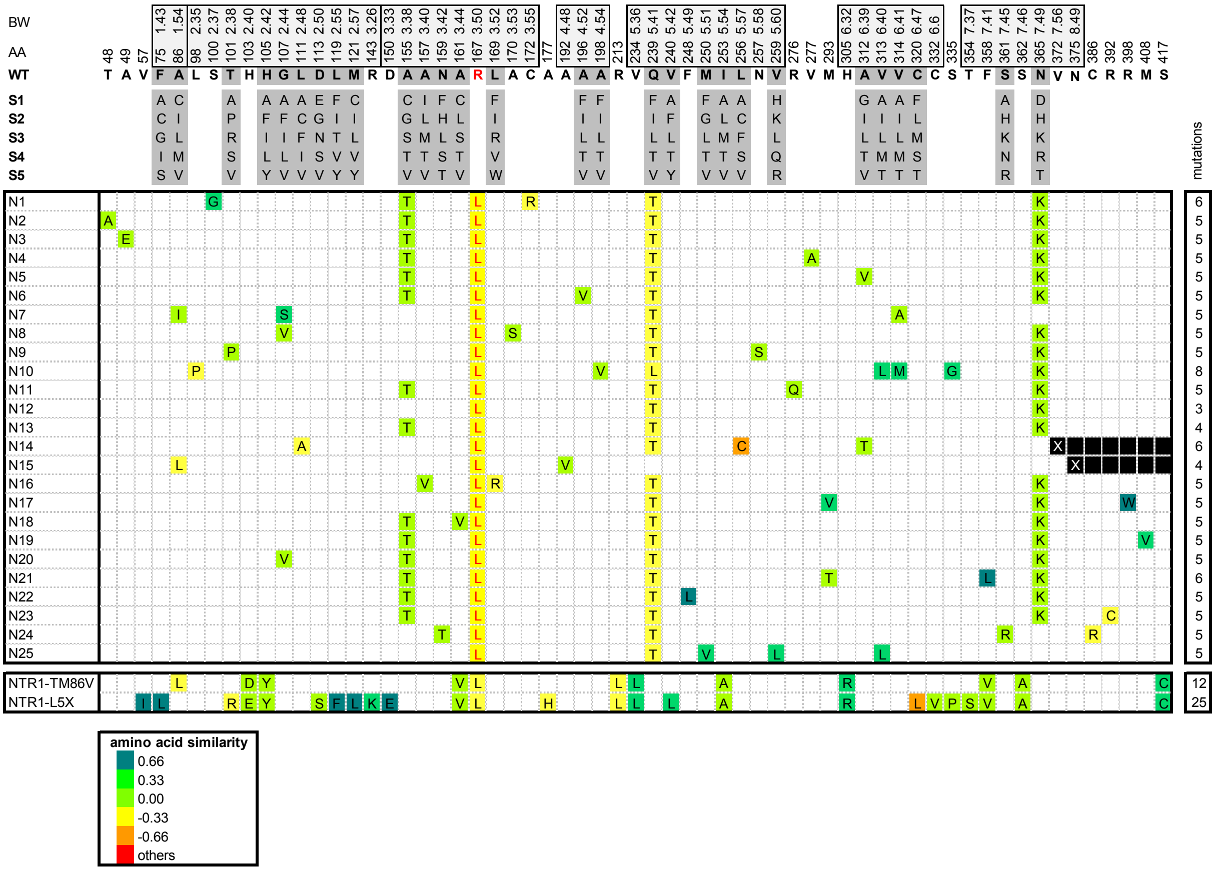

Supplementary Figure 3. Sequence representation of 25 selected NTR1 variants (N1–N25) and variants NTR1-TM86V and NTR1-L5X obtained from previous selections in *E. coli*[12]. Mutations are color-coded by sequence similarity to WT NTR1 using a Dayhoff matrix[8]. Randomized positions are shaded grey, and the fixed mutation R165L$^{3.50}$ is indicated by red lettering. Variants N14 and N15 contained single nucleotide deletions at the end of TM7 (depicted by an X), leading to a frame shift of the remaining sequence. Mutations per gene are given at the right. AA, residue number; BW, residue number according to Ballesteros-Weinstein; S1–S5, alternative residues used for randomization, at the respective position indicated.

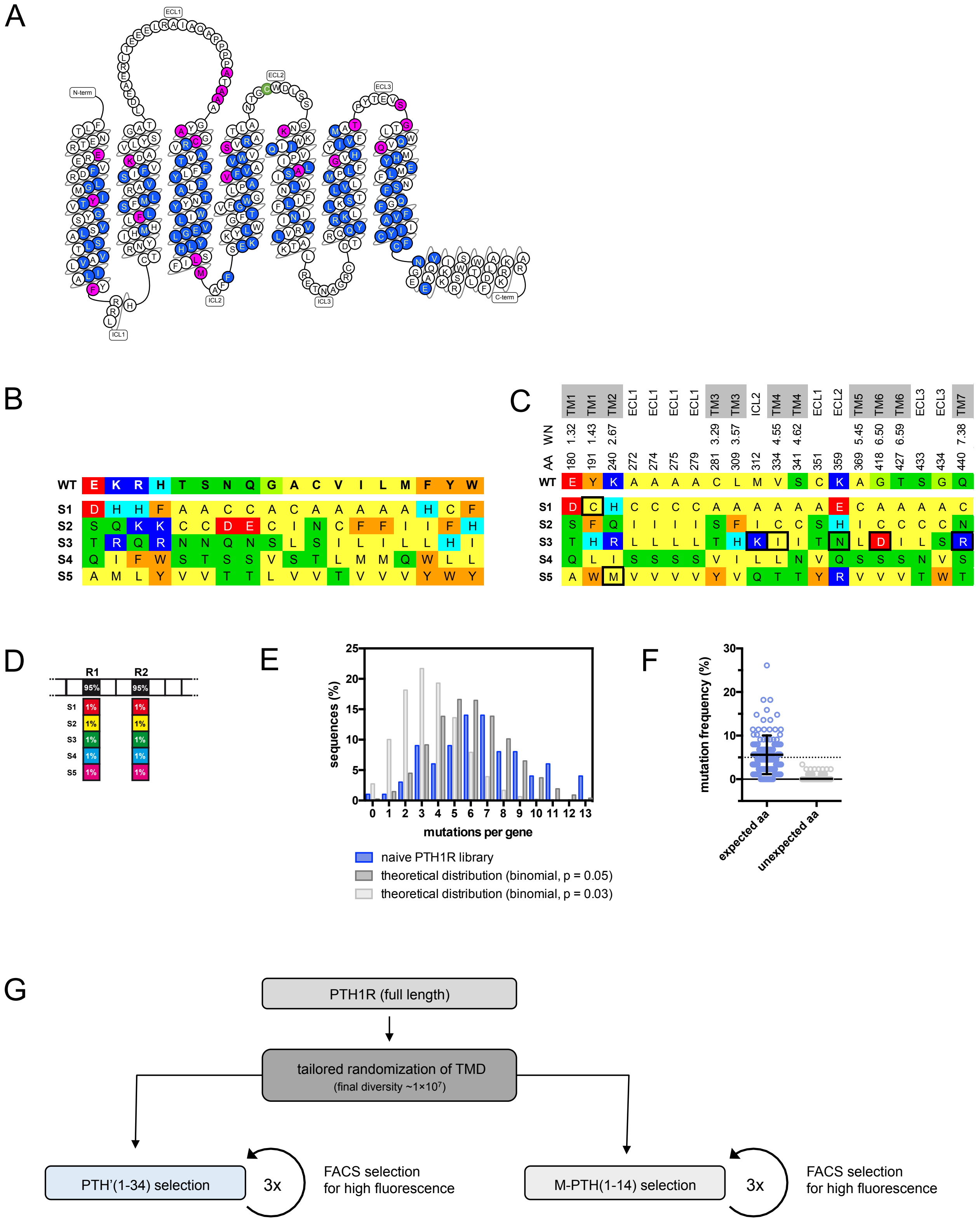

Supplementary Figure 4. Library design and selection strategy for PTH1R. (*A*) A homology model for PTH1R based on the crystal structure of glucagon receptor (PDB ID: 4L6R) was generated, and 99 residues within the TMD (residues 171–480) were selected for randomization (blue). Additionally, 19 residues identified in a previous evolution campaign in yeast[3] (Klenk et al. unpublished) (magenta) as well as the conserved Cys351 in ECL2 (green) were randomized. (*B*) Substitution matrix for 18 amino acid types. Asp and Pro were not among the WT residues assigned for randomization and thus are not contained in the matrix. Each of the five alternative residues for randomization (S1–S5) is based on amino acid similarity to the wild-type (WT) amino acid. (C) Yeast-derived residues and Cys351

were randomized following the same scheme as in (B) with the exception that stabilizing amino acids (black outline) were included in the substitution matrix. AA, residue number; WN, residue number according to Wootten[1]. (*D*) The mutation frequency was chosen to yield an average distribution of 3–5 mutations per gene. For this purpose, at each position, codons for five alternative amino acids (S1–S5) were incorporated with 1% frequency each. (*E–F*) Mutation analysis of naïve PTH1R library. Sequence analysis of 87 clones of the naïve library revealed an average distribution of 6.65 mutations per gene, corresponding to the expected theoretical mutation rate (*E*). At each position on average 5.59% residues corresponded to expected mutations (encoded by the nucleotide triplets) whereas 0.10% were unexpected mutations (i.e. mutations at constant positions or non-encoded mutations). Each data point depicts the frequency of expected and unexpected mutations at a specific position of PTH1R (amino-acids 179–480). Dotted line, overall anticipated mutation frequency. Error bars represent s.d. (*F*). (*E*) Selection scheme for PTH1R. After transferring the library into the *Vaccinia* vector, A-431 cells were infected and three consecutive selection rounds were performed either with 120 nM PTH'(1–34)-HL647 or with 120 nM M-PTH(1–14)-HL647.

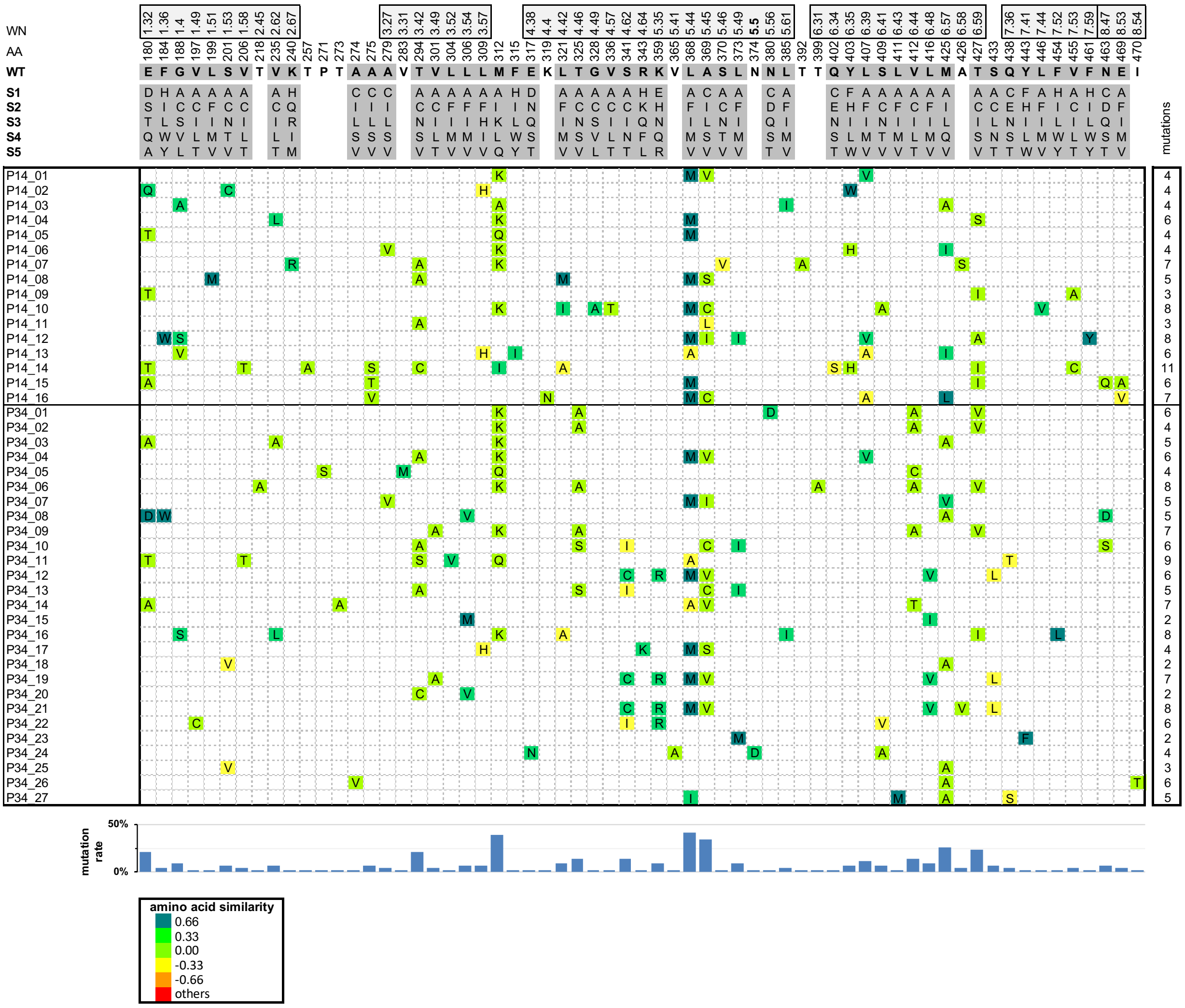

Supplementary Figure 5. Sequence representation of 43 selected PTH1R variants. Mutations are color-coded by sequence similarity to WT PTH1R using a Dayhoff matrix[8]. Mutation rate for each position is shown as a bar graph. Mutations per gene are given at the right. AA, residue number; WN, residue number according to Wootten[1]; S1–S5, alternative residues used for randomization at the respective position indicated.

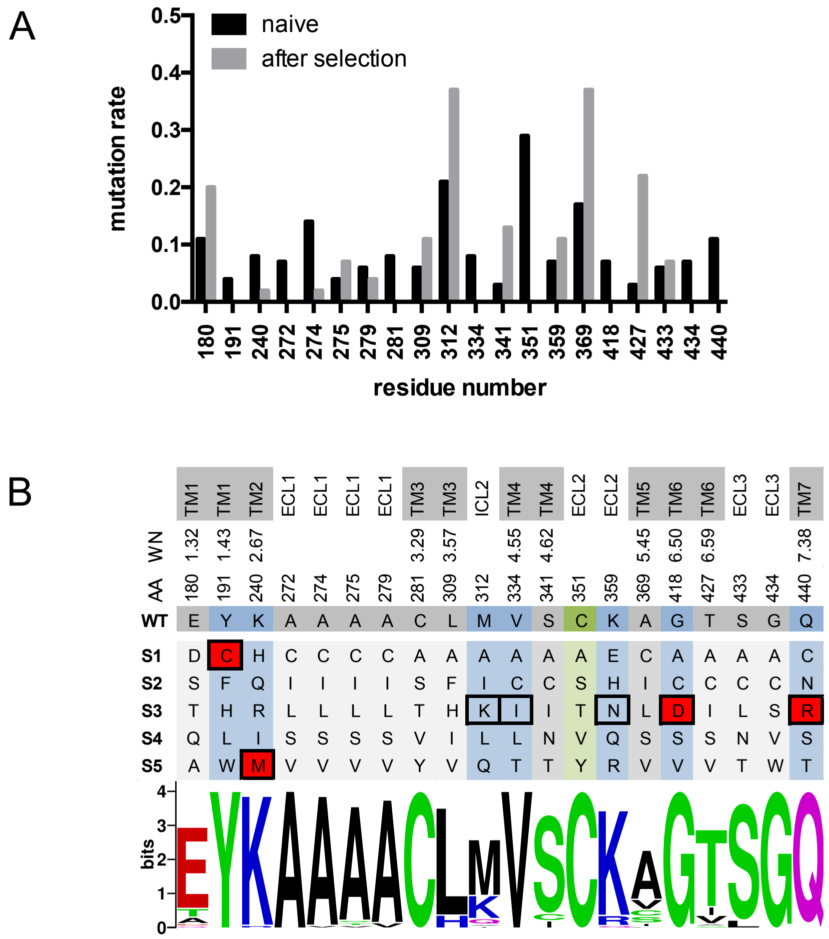

Supplementary Figure 6. Mutations that disrupt PTH1R signaling are deselected. (*A*) Mutation rate of the naive library and after three selection rounds. 96 sequences of each pool were analyzed. Shown are 19 positions, which had been derived from a previous yeast evolution campaign to stabilize PTH1R[3] (Klenk et al. unpublished), and position 351, which is required for disulfide bond formation between ECL2 and ECL3 in wild-type PTH1R[3] (c.f. **Supplementary Fig. 4A**). (*B*) Amino acid distribution of 92 clones after three selection rounds. The wild-type sequence (WT) and the initial randomization scheme (S1 to S5) (c.f. **Supplementary Fig. 4B–D**) are shown in the top panel. Stability-conferring positions identified in the yeast evolution campaign are shaded in blue and the respective stabilizing mutation is marked by a black outline. Stabilizing mutations Y191$^{1.43}$C, K240$^{2.67}$M, G418$^{6.50}$D and Q440$^{7.38}$R that disrupted receptor signaling[3] are shaded in red. C351 required for disulfide bond formation between ECL2 and ECL3 is shaded in green. The sequence logo shows the amino acid distribution of 92 clones after the selection at the positions indicated, indicating that receptor-inactivating mutations have been deselected. AA, residue number; WN, residue number according to Wootten[1]

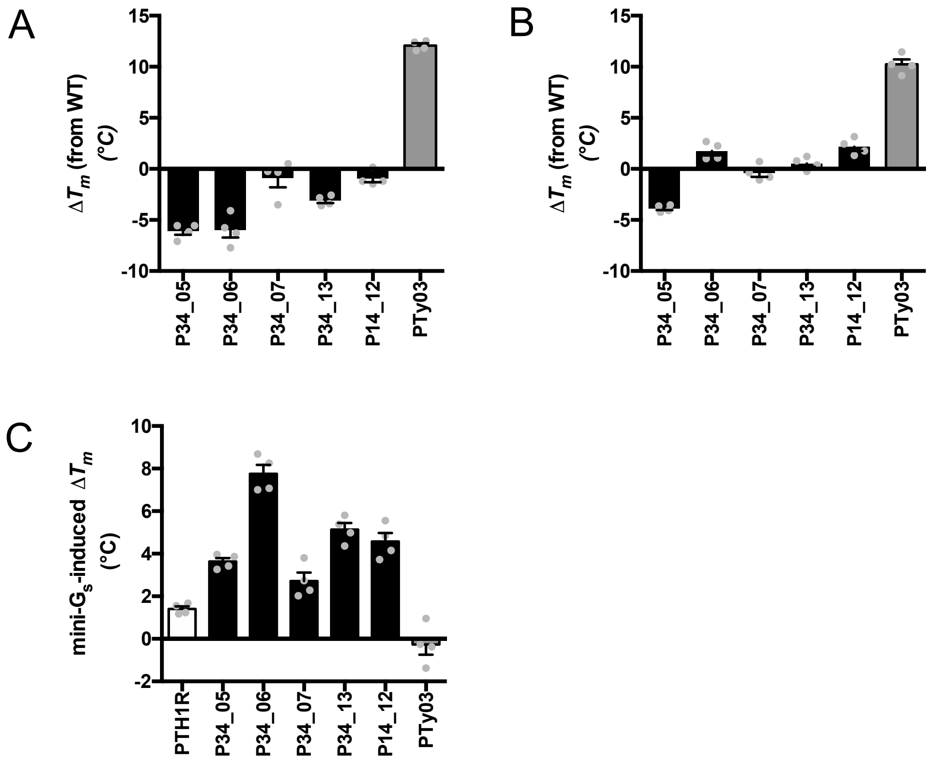

Supplementary Figure 7. Thermostability of evolved PTH1R variants is G protein-dependent. Thermostability of PTH1R variants was assessed in membrane preparations in the absence (*A*) or presence (*B*) of 12.5 µM mini-G_s. Data are given as the change in T_m from wild-type PTH1R. (*C*) Change in T_m induced by the presence of G protein. Data are shown as the change in T_m from each variant in absence of mini-G_s. The thermostabilized, signaling-inactive variant PTy03 (3) was included as control (grey). Data repesent mean values ± s.e.m. of 4 independent experiments (**Supplementary Table 5**).

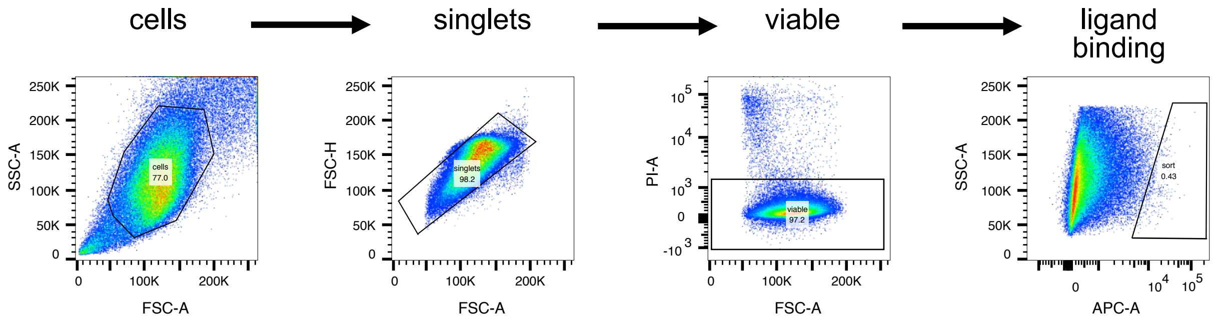

Supplementary Figure 8. Gating strategy for flow-cytrometric analyis and FACS. Cells were identified in a FSC-A/SSC-A scatter plot. Singlet cells were then gated via FSC-A/FSC-H. Viable cells were gated using propidium iodine (PI). For selections, cells with highest fluorescent levels in the APC channel were sorted.

Supplementary Table 1 | Pharmacological data of evolved NTR1 variants.

	expression	NT(8–13) binding	IP1 signaling	
	(fold of WT)	pIC_{50} (log M)	pEC_{50} (log M)	E_{max} (% WT)
N1	47.8 ± 10.7 (2)	8.35 ± 0.03 (3)	8.15 ± 0.13 (2)	7.0 ± 0.5 (2)
N2	54.2 ± 4.3 (2)	8.34 ± 0.04 (3)	8.25 ± 0.08 (2)	17.6 ± 0.7 (2)
N3	43.7 ± 2.2 (2)	8.51 ± 0.10 (3)	8.14 ± 0.07 (2)	17.2 ± 0.6 (2)
N4	51.1 ± 2.3 (2)	8.42 ± 0.04 (3)	8.24 ± 0.05 (2)	15.0 ± 0.4 (2)
N5	45.6 ± 1.5 (2)	8.43 ± 0.03 (3)	8.19 ± 0.09 (2)	15.9 ± 0.7 (2)
N6	49.5 ± 3.7 (2)	8.58 ± 0.06 (3)	8.34 ± 0.08 (2)	9.8 ± 0.4 (2)
N7	74.4 ± 7.1 (2)	8.11 ± 0.05 (3)	7.87 ± 0.09 (2)	7.1 ± 0.3 (2)
N8	76.4 ± 0.6 (2)	8.00 ± 0.05 (3)	7.83 ± 0.06 (2)	13.4 ± 0.5 (2)
N9	36.1 ± 2.0 (2)	8.27 ± 0.05 (3)	7.50 ± 0.40 (2)	3.2 ± 0.8 (2)
N10	40.2 ± 2.0 (2)	7.93 ± 0.08 (3)	8.31 ± 0.23 (2)	2.7 ± 0.3 (2)
N11	49.2 ± 8.6 (2)	8.35 ± 0.07 (3)	8.11 ± 0.06 (2)	15.7 ± 0.5 (2)
N12	36.0 ± 1.8 (2)	8.16 ± 0.08 (3)	8.13 ± 0.08 (2)	15.0 ± 0.6 (2)
N13	44.4 ± 5.0 (2)	8.37 ± 0.10 (3)	8.20 ± 0.07 (2)	17.7 ± 0.6 (2)
N14	82.5 ± 9.9 (3)	8.07 ± 0.03 (3)	n.a.	0.4 ± 0.2 (2)
N15	28.8 ± 8.3 (2)	8.20 ± 0.02 (3)	8.71 ± 0.34 (2)	1.4 ± 0.2 (2)
N16	30.8 ± 1.9 (2)	8.36 ± 0.07 (3)	8.18 ± 0.36 (2)	1.7 ± 0.3 (2)
N17	33.7 ± 6.8 (2)	8.25 ± 0.06 (3)	8.22 ± 0.08 (2)	11.3 ± 0.5 (2)
N18	48.3 ± 12.9 (2)	8.17 ± 0.08 (3)	8.09 ± 0.09 (2)	15.7 ± 0.7 (2)
N19	27.9 ± 2.7 (2)	8.32 ± 0.05 (3)	8.22 ± 0.18 (2)	17.0 ± 1.5 (2)
N20	25.3 ± 0.3 (2)	8.35 ± 0.02 (3)	8.17 ± 0.13 (2)	17.6 ± 1.1 (2)
N21	40.9 ± 1.7 (2)	8.49 ± 0.03 (3)	8.18 ± 0.18 (2)	5.7 ± 0.5 (2)
N22	27.6 ± 0.1 (2)	8.39 ± 0.07 (3)	8.26 ± 0.09 (2)	15.9 ± 0.7 (2)
N23	35.5 ± 4.1 (2)	8.21 ± 0.06 (3)	8.14 ± 0.08 (2)	33.4 ± 1.4 (2)
N24	41.0 ± 1.5 (2)	7.79 ± 0.09 (3)	8.16 ± 0.17 (2)	6.2 ± 0.5 (2)
N25	42.2 ± 1.0 (2)	7.98 ± 0.02 (3)	8.06 ± 0.09 (2)	5.0 ± 0.2 (2)
NTR1	1.0 ± 0.0 (3)	7.13 ± 0.07 (3)	7.95 ± 0.07 (2)	98.9 ± 1.8 (2)
NTR1_R167L	2.5 ± 0.8 (3)	7.57 ± 0.07 (3)	7.95 ± 0.07 (2)	34.4 ± 1.3 (2)
NTR1-TM86V	47.2 ± 6.5 (2)	n.d.	n.d.	n.d.
NTR1-L5X	33.4 ± 1.0 (2)	n.d.	n.d.	n.d.

Expression levels were determined by flow cytometry using 20 nM HL488-NT(8–13). IC_{50} values were derived from whole-cell ligand competition-binding experiments with NT(8–13). IP1 accumulation was measured in whole cells after stimulation with 0–500 nM NT(8–13). All data are represented as mean values ± s.e.m.. The number of experiments is given in parentheses. n.d., not determined; n.a. not applicable

Supplementary Table 2 | Thermostability of evolved NTR1 variants

	T_m (°C)
N8	60.0 ± 0.7 (4)
N12	58.1 ± 0.6 (4)
N13	56.4 ± 0.6 (4)
N14	62.4 ± 0.6 (4)
N15	59.8 ± 0.5 (4)
N21	57.7 ± 0.4 (4)
N23	57.9 ± 0.4 (4)
NTR1_R167L	51.4 ± 0.6 (4)
NTR1	52.5 ± 0.6 (4)

Thermostability data were obtained by measuring loss of ligand binding as a function of temperature in membrane fractions All data are represented as mean values ± s.e.m.. The number of independent experiments is given in parentheses.

Supplementary Table 3 | Expression and ligand binding of evolved PTH1R variants.

	expression (fold of WT)	M-PTH(1–14) binding pIC_{50} (log M)	PTH(1–34) binding pIC_{50} (log M)
P14_01	5.5 ± 1.5 (3)	7.86 ± 0.70 (5)	7.86 ± 0.09 (2)
P14_02	3.3 ± 0.4 (3)	7.19 ± 0.09 (3)	7.83 ± 0.08 (2)
P14_03	6.8 ± 2.1 (3)	7.83 ± 0.14 (3)	8.07 ± 0.11 (2)
P14_04	2.0 ± 0.3 (3)	7.26 ± 0.02 (3)	7.73 ± 0.01 (2)
P14_05	5.2 ± 2.5 (2)	7.14 ± 0.02 (3)	7.82 ± 0.20 (4)
P14_06	2.3 ± 0.6 (3)	7.28 ± 0.01 (3)	7.32 ± 0.03 (2)
P14_07	1.4 ± 0.2 (3)	7.77 ± 0.06 (3)	8.40 ± 0.18 (2)
P14_08	2.1 ± 0.4 (3)	7.58 ± 0.02 (3)	8.32 ± 0.17 (2)
P14_09	1.7 ± 0.5 (3)	7.50 ± 0.11 (3)	7.82 ± 0.01 (2)
P14_10	3.2 ± 1.0 (3)	7.79 ± 0.09 (4)	8.05 ± 0.11 (2)
P14_11	3.3 ± 0.8 (2)	7.06 ± 0.15 (5)	7.98 ± 0.03 (2)
P14_12	3.6 ± 0.7 (2)	7.25 ± 0.50 (4)	8.09 ± 0.06 (2)
P14_13	3.3 ± 1.2 (3)	6.83 ± 0.49 (3)	8.31 ± 0.08 (2)
P14_14	1.5 ± 0.4 (3)	7.85 ± 0.20 (3)	8.32 ± 0.07 (2)
P14_15	2.7 ± 0.9 (3)	7.67 ± 0.12 (3)	8.06 ± 0.01 (2)
P14_16	1.8 ± 0.7 (3)	7.85 ± 0.33 (3)	8.50 ± 0.24 (2)
P34_01	6.7 ± 2.2 (3)	7.46 ± 0.04 (3)	7.97 ± 0.02 (2)
P34_02	7.5 ± 2.6 (3)	7.59 ± 0.06 (6)	7.95 ± 0.04 (2)
P34_03	5.9 ± 1.5 (3)	7.02 ± 0.06 (4)	7.69 ± 0.04 (2)
P34_04	8.5 ± 2.3 (2)	7.70 ± 0.06 (3)	8.04 ± 0.16 (2)
P34_05	4.5 ± 1.4 (3)	7.31 ± 0.05 (3)	7.89 ± 0.12 (2)
P34_06	5.9 ± 1.6 (3)	8.55 ± 1.12 (5)	8.07 ± 0.10 (2)
P34_07	9.1 ± 3.8 (3)	7.74 ± 0.07 (3)	7.99 ± 0.10 (2)
P34_08	2.6 ± 0.3 (3)	7.62 ± 0.13 (3)	7.90 ± 0.01 (2)
P34_09	4.6 ± 1.5 (3)	7.88 ± 0.07 (3)	7.95 ± 0.06 (2)
P34_10	3.7 ± 1.3 (3)	7.23 ± 0.03 (3)	8.09 ± 0.16 (2)
P34_11	2.9 ± 0.8 (3)	7.01 ± 0.04 (3)	7.83 ± 0.10 (2)
P34_12	3.6 ± 1.7 (3)	7.58 ± 0.08 (3)	8.07 ± 0.26 (2)
P34_13	4.7 ± 1.8 (3)	7.34 ± 0.07 (3)	7.87 ± 0.10 (2)
P34_14	1.4 ± 0.1 (3)	7.46 ± 0.07 (5)	8.09 ± 0.03 (2)
P34_15	1.3 ± 0.2 (3)	7.11 ± 0.05 (4)	7.98 ± 0.06 (3)
P34_16	1.4 ± 0.2 (3)	7.58 ± 0.10 (5)	7.84 ± 0.03 (2)
P34_17	1.4 ± 0.1 (3)	7.86 ± 0.15 (3)	8.28 ± 0.03 (3)
P34_18	4.1 ± 1.6 (3)	7.66 ± 0.03 (4)	8.56 ± 0.00 (2)
P34_19	5.3 ± 2.3 (3)	7.63 ± 0.12 (3)	8.40 ± 0.00 (2)
P34_20	2.9 ± 1.3 (3)	7.62 ± 0.06 (5)	8.33 ± 0.02 (2)
P34_21	2.3 ± 0.2 (2)	6.99 ± 0.03 (3)	7.85 ± 0.04 (2)
P34_22	1.4 ± 0.2 (3)	7.58 ± 0.06 (3)	8.44 ± 0.15 (2)
P34_23	2.2 ± 0.7 (2)	7.51 ± 0.06 (3)	8.17 ± 0.01 (2)
P34_24	0.9 ± 0.0 (2)	6.25 ± 0.10 (3)	8.27 ± 0.08 (2)
P34_25	1.3 ± 0.4 (3)	8.28 ± 0.49 (3)	8.60 ± 0.20 (2)

P34_26	1.4 ± 0.5 (3)	7.17 ± 0.60 (3)	8.40 ± 0.05 (2)
P34_27	4.1 ± 1.8 (3)	4.86 ± 1.31 (5)	8.11 ± 0.12 (2)
PTH1R	1.0 ± 0.0 (2)	6.23 ± 0.08 (7)	7.80 ± 0.09 (8)

Expression levels were determined by flow cytometry analysis using PTH'(1–34)-HL647. HTRF-ligand binding assays were performed on whole cells. M-PTH(1–14) binding was determined in constructs only containing the TMD of receptor whereas PTH(1–34) binding was obtained in full-length receptor constructs. All data are represented as mean values ± s.e.m.. The number of independent experiments is given in parentheses.

Supplementary Table 4 | cAMP accumulation of evolved PTH1R variants

	**pEC_{50} (log M)**	E_{max} **(fold of WT)**
P14_01	9.37 ± 0.05 (4)	1.19 ± 0.23 (4)
P14_02	10.42 ± 0.36 (3)	1.06 ± 0.17 (3)
P14_03	9.89 ± 0.18 (4)	1.24 ± 0.22 (4)
P14_04	10.31 ± 0.01 (3)	1.86 ± 0.50 (3)
P14_05	10.75 ± 0.21 (5)	0.79 ± 0.23 (5)
P14_06	10.48 ± 0.48 (3)	0.87 ± 0.33 (3)
P14_07	10.51 ± 0.10 (3)	0.95 ± 0.28 (3)
P14_08	10.22 ± 0.09 (3)	1.41 ± 0.58 (3)
P14_09	10.01 ± 0.08 (2)	1.85 ± 0.65 (2)
P14_10	9.84 ± 0.27 (3)	0.57 ± 0.12 (3)
P14_11	10.32 ± 0.04 (2)	1.10 ± 0.25 (2)
P14_12	10.42 ± 0.50 (2)	0.57 ± 0.14 (2)
P14_13	9.79 ± 0.44 (2)	1.00 ± 0.14 (2)
P14_14	10.34 ± 0.35 (2)	0.72 ± 0.16 (2)
P14_15	9.22 ± 0.31 (2)	1.10 ± 0.21 (2)
P14_16	9.79 ± 0.18 (2)	1.06 ± 0.22 (2)
P34_01	10.30 ± 0.43 (3)	1.12 ± 0.28 (3)
P34_02	10.13 ± 0.06 (6)	1.39 ± 0.31 (6)
P34_03	10.25 ± 0.43 (3)	1.74 ± 0.35 (3)
P34_04	9.93 ± 0.17 (3)	1.61 ± 0.28 (3)
P34_05	10.13 ± 0.46 (3)	1.55 ± 0.28 (3)
P34_06	10.31 ± 0.32 (3)	1.55 ± 0.25 (3)
P34_07	9.22 ± 0.08 (3)	1.49 ± 0.42 (3)
P34_08	10.60 ± 0.47 (4)	1.58 ± 0.92 (4)
P34_09	10.53 ± 0.23 (2)	1.64 ± 0.19 (2)
P34_10	10.82 ± 0.48 (2)	1.75 ± 0.01 (2)
P34_11	10.17 ± 0.34 (2)	1.81 ± 0.46 (2)
P34_12	9.79 ± 0.41 (2)	1.48 ± 0.19 (2)
P34_13	10.93 ± 0.40 (2)	2.17 ± 0.66 (2)
P34_14	10.21 ± 0.10 (2)	1.43 ± 0.29 (2)
P34_15	10.04 ± 0.24 (5)	1.24 ± 0.28 (5)
P34_16	10.95 ± 0.21 (3)	1.11 ± 0.05 (3)
P34_17	8.88 ± 1.53 (3)	2.16 ± 0.32 (3)
P34_18	10.48 ± 0.33 (4)	0.82 ± 0.06 (4)
P34_19	10.43 ± 0.17 (2)	0.61 ± 0.08 (2)
P34_20	10.24 ± 0.39 (2)	0.83 ± 0.13 (2)
P34_21	9.73 ± 0.11 (2)	1.24 ± 0.06 (2)
P34_22	9.72 ± 0.01 (2)	0.32 ± 0.13 (2)
P34_23	10.50 ± 0.19 (2)	0.46 ± 0.10 (2)
P34_24	10.06 ± 0.41 (5)	1.00 ± 0.04 (5)
P34_25	10.02 ± 0.30 (2)	0.90 ± 0.34 (2)

P34_26	10.10 ± 0.37 (2)	0.86 ± 0.32 (2)
P34_27	9.90 ± 0.05 (3)	0.81 ± 0.11 (3)
PTH1R	10.49 ± 0.13 (5)	1.00 ± 0.00 (5)

cAMP accumulation was measured in transiently transfected HEK293T cells after stimulation with 0–1 μM PTH(1–34). All data are represented as mean values ± s.e.m.. The number of independent experiments is given in parentheses.

Supplementary Table 5 | Thermostability of evolved PTH1R variants

	T_m (°C)	
	**- mini-G$_s$**	**+ mini-G$_s$**
P34_05	44.7 ± 0.2 (4)	48.3 ± 0.2 (4)
P34_06	46.2 ± 0.5 (4)	53.8 ± 0.4 (4)
P34_07	48.9 ± 0.8 (4)	51.6 ± 0.4 (4)
P34_13	47.5 ± 0.3 (4)	52.7 ± 0.3 (4)
P14_12	49.7 ± 0.4 (4)	54.5 ± 0.4 (4)
PTH1R	50.6 ± 1.0 (4)	52.0 ± 0.1 (4)
PTy03	62.7 ± 0.3 (4)	62.7 ± 0.5 (4)

Thermostability data were obtained by measuring loss of ligand binding as a function of temperature in membrane fractions in the absence or presence of 12.5 μM mini-G$_s$. All data are represented as mean values ± s.e.m.. The number of independent experiments is given in parentheses.

Supplementary References

1. Wootten, D., Simms, J., Miller, L. J., Christopoulos, A. & Sexton, P. M. Polar transmembrane interactions drive formation of ligand-specific and signal pathway-biased family B G protein-coupled receptor conformations. *Proc. Natl. Acad. Sci. U. S. A.* **110,** 5211–5216 (2013).
2. Sarkar, C. A. *et al.* Directed evolution of a G protein-coupled receptor for expression, stability, and binding selectivity. *Proc. Natl. Acad. Sci. U. S. A.* **105,** 14808–14813 (2008).
3. Ehrenmann, J. *et al.* High-resolution crystal structure of parathyroid hormone 1 receptor in complex with a peptide agonist. *Nat. Struct. Mol. Biol.* **25,** 1086–1092 (2018).
4. Dodevski, I. & Plückthun, A. Evolution of three human GPCRs for higher expression and stability. *J. Mol. Biol.* **408,** 599–615 (2011).
5. Schütz, M. *et al.* Directed evolution of G protein-coupled receptors in yeast for higher functional production in eukaryotic expression hosts. *Sci. Rep.* **6,** 21508 (2016).
6. Waltenspühl, Y., Jeliazkov, J. R., Kummer, L. & Plückthun, A. Directed evolution for high functional production and stability of a challenging G protein-coupled receptor. *Sci. Rep.* **11,** 8630 (2021).
7. Henikoff, S. & Henikoff, J. G. Amino acid substitution matrices from protein blocks. *Proc. Natl. Acad. Sci. U. S. A.* **89,** 10915–10919 (1992).
8. Dayhoff, M. O., Schwartz, R. M. & Orcutt, B. C. in *Atlas of Protein Sequence and Structure* (ed. Dayhoff, M. O.) **5,** 345–352 (National Biomedical Research Foundation Silver Spring MD, 1978).
9. Rios, S. *et al.* GPCRtm: An amino acid substitution matrix for the transmembrane region of class A G Protein-Coupled Receptors. *BMC Bioinformatics* **16,** 206 (2015).
10. Van den Brulle, J. *et al.* A novel solid phase technology for high-throughput gene synthesis. *BioTechniques* **45,** 340–343 (2008).
11. Zhai, W. *et al.* Synthetic antibodies designed on natural sequence landscapes. *J. Mol. Biol.* **412,** 55–71 (2011).
12. Schlinkmann, K. M. *et al.* Maximizing detergent stability and functional expression of a GPCR by exhaustive recombination and evolution. *J. Mol. Biol.* **422,** 414–428 (2012).
