# Supplementary material for: A Vaccinia-based system for directed evolution of GPCRs in mammalian cells
Source: Nat Commun. 2023 Mar 30;14:1770. doi: 10.1038/s41467-023-37191-8 (PMC10063554; doi:10.1038/s41467-023-37191-8)
Supplement: Supplementary file 3 — Reporting Summary [file 41467_2023_37191_MOESM3_ESM.pdf]

nature portfolio

Corresponding author(s): Andreas Plückthun, PhD
Christoph Klenk, MD PhD

Last updated by author(s): Feb 24, 2023

Reporting Summary

Nature Portfolio wishes to improve the reproducibility of the work that we publish. This form provides structure for consistency and transparency in reporting. For further information on Nature Portfolio policies, see our Editorial Policies and the Editorial Policy Checklist.

Statistics

For all statistical analyses, confirm that the following items are present in the figure legend, table legend, main text, or Methods section.

n/a	Confirmed	
☐	☒	The exact sample size (*n*) for each experimental group/condition, given as a discrete number and unit of measurement
☐	☒	A statement on whether measurements were taken from distinct samples or whether the same sample was measured repeatedly
☐	☒	The statistical test(s) used AND whether they are one- or two-sided<br>*Only common tests should be described solely by name; describe more complex techniques in the Methods section.*
☒	☐	A description of all covariates tested
☒	☐	A description of any assumptions or corrections, such as tests of normality and adjustment for multiple comparisons
☐	☒	A full description of the statistical parameters including central tendency (e.g. means) or other basic estimates (e.g. regression coefficient) AND variation (e.g. standard deviation) or associated estimates of uncertainty (e.g. confidence intervals)
☐	☒	For null hypothesis testing, the test statistic (e.g. *F*, *t*, *r*) with confidence intervals, effect sizes, degrees of freedom and *P* value noted<br>*Give P values as exact values whenever suitable.*
☒	☐	For Bayesian analysis, information on the choice of priors and Markov chain Monte Carlo settings
☒	☐	For hierarchical and complex designs, identification of the appropriate level for tests and full reporting of outcomes
☒	☐	Estimates of effect sizes (e.g. Cohen's *d*, Pearson's *r*), indicating how they were calculated

Our web collection on statistics for biologists contains articles on many of the points above.

Software and code

Policy information about availability of computer code

Data collection	Microsoft Excel V 2108, WebLogo V2.8.2, BD FACSDIVA V 6.0, GPCRdb.org
Data analysis	Microsoft Excel V 2108, Graphpad Prism V 6.07, FlowJo V 10, CLC Workbench V22.0.2, PyMOL V1.8.2

For manuscripts utilizing custom algorithms or software that are central to the research but not yet described in published literature, software must be made available to editors and reviewers. We strongly encourage code deposition in a community repository (e.g. GitHub). See the Nature Portfolio guidelines for submitting code & software for further information.

Data

Policy information about availability of data

All manuscripts must include a data availability statement. This statement should provide the following information, where applicable:

- Accession codes, unique identifiers, or web links for publicly available datasets
- A description of any restrictions on data availability
- For clinical datasets or third party data, please ensure that the statement adheres to our policy

The following protein structures were used in this manuscript:
High Resolution Structure of Thermostable Agonist-bound Neurotensin Receptor 1 Mutant without Lysozyme Fusion, PBD 4BUO
Structure of the class B human glucagon G protein coupled receptor, PDB 4L6R
Protein sequence alignments from 296 class A GPCRs were obtained from GPCRdb (www.gpcrdb.org).

All data needed to evaluate the conclusions in the paper are present in the paper and/or in the Supplementary Information. Source data are provided with this paper. Additional data related to this paper may be requested from the authors upon reasonable request. Source data are provided with this paper.

Human research participants

Policy information about studies involving human research participants and Sex and Gender in Research.

Reporting on sex and gender	n/a
Population characteristics	n/a
Recruitment	n/a
Ethics oversight	n/a

Note that full information on the approval of the study protocol must also be provided in the manuscript.

Field-specific reporting

Please select the one below that is the best fit for your research. If you are not sure, read the appropriate sections before making your selection.

- [x] Life sciences
- [ ] Behavioural & social sciences
- [ ] Ecological, evolutionary & environmental sciences

For a reference copy of the document with all sections, see nature.com/documents/nr-reporting-summary-flat.pdf

Life sciences study design

All studies must disclose on these points even when the disclosure is negative.

Sample size	No sample size calculation was performed. The study reports biochemical assays, which have been done in the number of independent experiments (independent transfections and readings) indicated in the text. Each experiment was measured in technical duplicates. For flow cytometry experiments, at least 3x10^4 cells were recorded. Based on our experience and on other studies with similiar methodology this number is large enough to identify individual experimental variations.
Data exclusions	No data were excluded.
Replication	The biochemical assays were carried out at least two times as described in the methods section, figure legends or tables. All attempts at replication were successful.
Randomization	This study is entirely based on in cellulo experiments. Thus, no allocation into experimental groups was needed which could be randomized.
Blinding	Investigators were not blinded during data collection or analysis. This was not possible since, transfection of the cells was conducted by the same investigators.

Reporting for specific materials, systems and methods

We require information from authors about some types of materials, experimental systems and methods used in many studies. Here, indicate whether each material, system or method listed is relevant to your study. If you are not sure if a list item applies to your research, read the appropriate section before selecting a response.

Materials & experimental systems

n/a	Involved in the study	
x		Antibodies
	x	Eukaryotic cell lines
x		Palaeontology and archaeology
x		Animals and other organisms
x		Clinical data
x		Dual use research of concern

Methods

n/a	Involved in the study	
x		ChIP-seq
	x	Flow cytometry
x		MRI-based neuroimaging

nature portfolio | reporting summary

Eukaryotic cell lines

Policy information about cell lines and Sex and Gender in Research

Cell line source(s)	HEK293T cells, A-431 and BS-C-1 cells: ATCC; CHO-S: Life Technologies
Authentication	No authentication was used, as the experiments depend only on transient transfection of a recombinant protein.
Mycoplasma contamination	All cell lines were mycoplasma free as monitored by regular tests.
Commonly misidentified lines (See ICLAC register)	No commonly misidentified cell lines were used in the study.

Flow Cytometry

Plots

Confirm that:

- [x] The axis labels state the marker and fluorochrome used (e.g. CD4-FITC).
- [x] The axis scales are clearly visible. Include numbers along axes only for bottom left plot of group (a 'group' is an analysis of identical markers).
- [x] All plots are contour plots with outliers or pseudocolor plots.
- [x] A numerical value for number of cells or percentage (with statistics) is provided.

Methodology

Sample preparation	Infected A-431 or transfected HEK293T cells were harvested using Accutase, pelleted and washed in PBS, 1% (w/v) BSA or Tris buffer [20 mM Tris-HCl (pH 7.4), 118 mM NaCl, 5.6 mM glucose, 1.2 mM KH2PO4, 1.2 mM MgSO4, 4.7 mM KCl, 1.8 mM CaCl, 0.1% (w/v) BSA]. Cells were resuspended at 2x10exp6 – 4x10exp7 cells per ml in FACS buffer or Tris buffer and incubated with fluorescent ligand on ice for 1 – 2 hours. To confirm specificity, duplicate cell samples were also incubated with a 100-fold excess of unlabeled ligand. Cells were then washed in the appropriate buffer and fixed in 0.5% paraformaldehyde with propidium iodide for live/ dead cell discrimination before analysis on the flow cytometer.
Instrument	BD FACSCanto 2, BD FACS Aria III
Software	BD FACSDiva V6.0
Cell population abundance	Cell population abundance is not relevant as homogenous cell lines were used (transfected/ infected cells).
Gating strategy	cell populations were identified by FSC/SSC gating and singlet cells were gated by FSC-A/FSC-W gating. For selections by FACS, the top 0.06-0.3% fluorescent cells in an FSC/APC gate were selected.

- [x] Tick this box to confirm that a figure exemplifying the gating strategy is provided in the Supplementary Information.
